# Health seeking behavior and use of medicinal plants among the Hamer ethnic group, South Omo zone, southwestern Ethiopia

**DOI:** 10.1186/s13002-016-0107-x

**Published:** 2016-10-06

**Authors:** Biniam Paulos, Teferi Gedif Fenta, Daniel Bisrat, Kaleab Asres

**Affiliations:** 1 Department of Pharmacy, Wollega University, P. O Box 395, Nekemte, Ethiopia; 2 Department of Pharmaceutics and Social Pharmacy, School of Pharmacy, College of Health Sciences, Addis Ababa University, P. O Box 1176, Addis Ababa, Ethiopia; 3 Department of Pharmaceutical Chemistry and Pharmacognosy, School of Pharmacy, College of Health Sciences, Addis Ababa University, P. O Box 1176, Addis Ababa, Ethiopia

**Keywords:** Ethnopharmacological information, Cross-sectional study, Focus group discussions, Semi-structured questionnaires, Hamer ethnic group, Southwestern Ethiopia

## Abstract

**Background:**

Health seeking behavior of people around the globe is affected by different socio-cultural and economic factors. In Ethiopia, people living in rural areas in particular, are noted for their use of medicinal plants as a major component of their health care option. This study was conducted to document ethnopharmacological information of the Hamer semi-pastoralists ethnic group in southwestern Ethiopia.

**Methods:**

A cross-sectional study was carried out whereby information on demographic characteristics, prevalence of perceived illnesses, factors associated with preference of health care seeking options, medicinal plants used and hoarded as well as some healers’ socio-economic characteristics were collected using two sets of semi-structured questionnaires – one for household (HH) heads and the other for traditional healers supplemented by focus group discussions (FGDs). Households were selected using a cluster sampling followed by systematic sampling techniques; whereas healers and FGD participants were purposively selected with the assistance of local leaders and elders from the community.

**Results:**

The study revealed that the use of traditional medicine among the Hamer ethnic group is very high. Females preferred traditional medicine more than males. The main reasons for this preference include effectiveness, low cost and ease of availability. Malaria (*gebeze*) was the most frequently occurring illness in the area identified by all FGD participants. A total of 60 different medicinal plants were reported [34 by HH respondents, 14 by traditional healers and 12 by both]. Fifty-one medicinal plants were fully identified, 3 at generic level and 6 have not yet been identified.

**Conclusion:**

It can be concluded that traditional medical practices, particularly herbal aspect, is widely used by the Hamer ethnic group, although health seeking behavior of the community is affected by different socio-economic and cultural factors.

**Electronic supplementary material:**

The online version of this article (doi:10.1186/s13002-016-0107-x) contains supplementary material, which is available to authorized users.

## Background

The use of natural products as medicinal agents dates back to prehistoric period [1]. Traditional medicine (TM) refers to health practices, approaches, knowledge and beliefs incorporating plant, animal and mineral-based medicines, spiritual therapies, manual techniques and exercises, applied singularly or in combination, to treat, diagnose, prevent illnesses or maintain well-being [2].

In Ethiopia, medicinal plants have been used to treat different diseases for many centuries, and religious and secular pharmacopoeias had been compiled since the 15^th^ century, resulting in medical pluralism [3, 4]. The studies of the tribal indigenous knowledge of plants and their local use is often linked to purpose-specific characteristics of plants, mainly, their efficacy to correct harmful symptoms or eliminate causal factors associated with particular conditions constituting an important but preliminary aspect of ethnopharmacological research [5].

Comparing the existence of the variety of cultures and diversity of climatic conditions, the documentation of ethnomedical use of plants is limited in Ethiopia [6]. Furthermore, most of the sources for these documentations focused only on the herbalists and the Ethiopian medico-religious manuscripts without giving due attention to the rich traditional knowledge and practices of ordinary people [6]. This trend might hinder access to the traditional knowledge preserved by grassroots; affecting the scope and quality of the documentation and the research on medicinal plants [7]. This is particularly true among pastoralist communities of Ethiopia where access to biomedical practitioners is limited. Thus, the purpose of this study is to assess and document traditional medicinal plants knowledge and uses among the Hamer ethnic group in South Omo zone, Southwestern Ethiopia.

## Methods

### Study area and socio-economic settings

Hamer woreda is one of the nine woredas (second from lowest administrative units in government structure) in South Omo Zone, Southern Nations, Nationalities and Peoples Region (SNNPR), with an estimated area of 731,565 hectares. It is located at 770 km to the southwest of Addis Ababa or 540 km from Hawassa, the capital of SNNPR. It is bordered by Bena-Tsemay to the north, Kenya-Kuraz-Borena of Oromia to the south, by Bena-Tsemay and of Borena of Oromia to the east and Kuraz woreda to the west [8]. Dimeka is the capital of Hamer woreda. The total population of the woreda is 59,160 (29,466 female and 29,694 male). Eighty percent of the population belongs to the Hamer ethnic group; 11.2 % to the Erbore ethnic group; and 2.47 % to the Kara ethnic group. A total of 3210 people live in Dimeka and the neighboring Turmi towns and the remaining 55,950 live in rural areas [8]. At the time of this survey, the woreda had three health centers and eight health posts. Harmful traditional practices and low coverage of health services resulted in low health status of the population in the woreda [8, 9].

### Sampling, data collection and analysis

Ethical approval was secured from the Institutional Ethics Review Board of the School of Pharmacy, Addis Ababa University, prior to starting of the study. Information on demographic characteristics, prevalence of perceived illnesses, factors associated with preference of health care seeking options, medicinal plants used and hoarded as well as some healers’ socio-economic characteristics were collected using two sets of semi-structured questionnaires – one for household (HH) heads and the other for traditional healers (Additional file 1) .

**Fig. 1:**
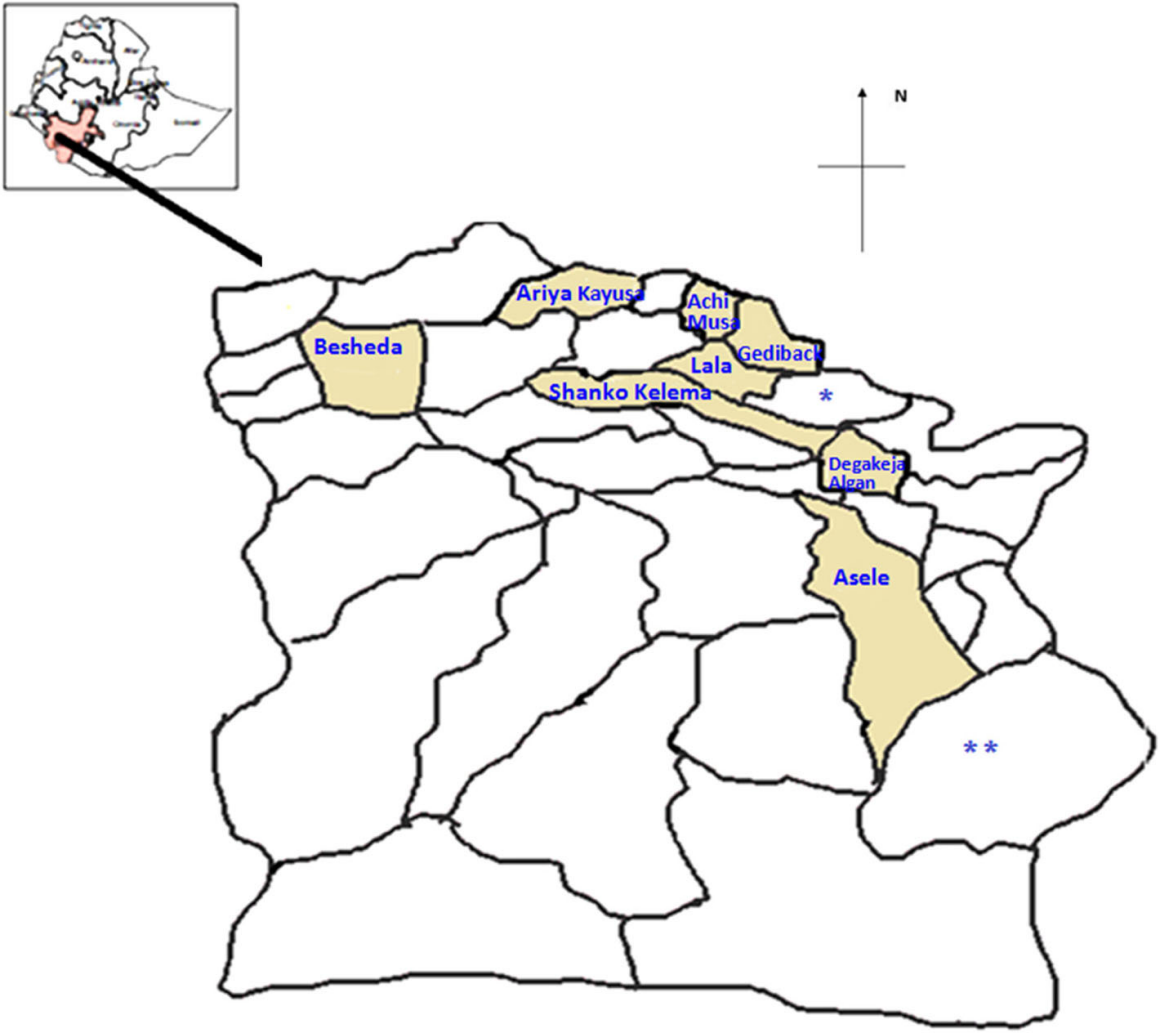
Map of Hamer Woreda (* Kebele has been moved to the adjacent Bena Tsenai woreda; ** Kebele has been merged with the neighbouring four kebeles)

Hamer woreda has 35 kebeles (lowest administrative unit) (Fig. 1). Eight kebeles were selected by simple random sampling. To select the final sampling unit, first cluster of HHs were selected randomly followed by systematically selecting specific HHs. A total of 1600 respondents, 200 HHs from each kebele, were included in the study. Since HHs were final sampling units for the HH survey, the respondents included the head of the house (husband) or the wife or in the absence of both, any members of the family who were above 18 years of age.

Eight key informant traditional healers were selected purposively based on their healing experiences as testified by community leaders, kebele administrators and community elders (Additional file 1). Focus group discussions (FGDs) were held in each selected kebele whereby each FGD group consisted of seven members selected from elders of the community whose ages ranged from 40 to 70 years.

Data collectors, who were high school students with good knowledge of the local and English languages, were given training for two days on the data collection instruments. Oral consent was obtained from each respondent before conducting the interview. Moreover, participants of the study have consented to their photograph being taken for publication, if necessary. Variables like socio-demographic characteristics of HH respondents, HH size, existence of illness during the 2 weeks preceding interview date, choice of treatment options, names and parts of plants used, etc. were entered in Statistical Package for the Social Sciences (SPSS) and analyzed. The qualitative data was analyzed thematically.

## Results

### Summary of FGDs

The FGDs were held in the following eight kebeles of the woreda: *Ariya Kayusa*, *Achi Musa*, *Besheda, Shanko Kelema, Gediback, Asele, Lala* and *Degakeja Algan*. Age of the participants ranged from 40 to 70 years (27 females and 29 males). The results of the FGDs were summarized by giving the local names of illnesses in italics. Major signs and symptoms or closer meanings of the illnesses are shown in Additional file 1.

Malaria (*gebeze*) was the most frequently occurring illness in the area identified by all FGD participants. In addition, eye diseases (*afo burka*), diarrhoea (*zen*), tinea infections (*berdate*), common cold (*gulfadhana*), evil eye (*chaqi*), jaundice (*ara*), skin disorders (*bishi/shelofecha*), snake bite (*guni*) and hypertension (*lognagena*) were identified as common illnesses that threaten the community.

**Fig. 2:**
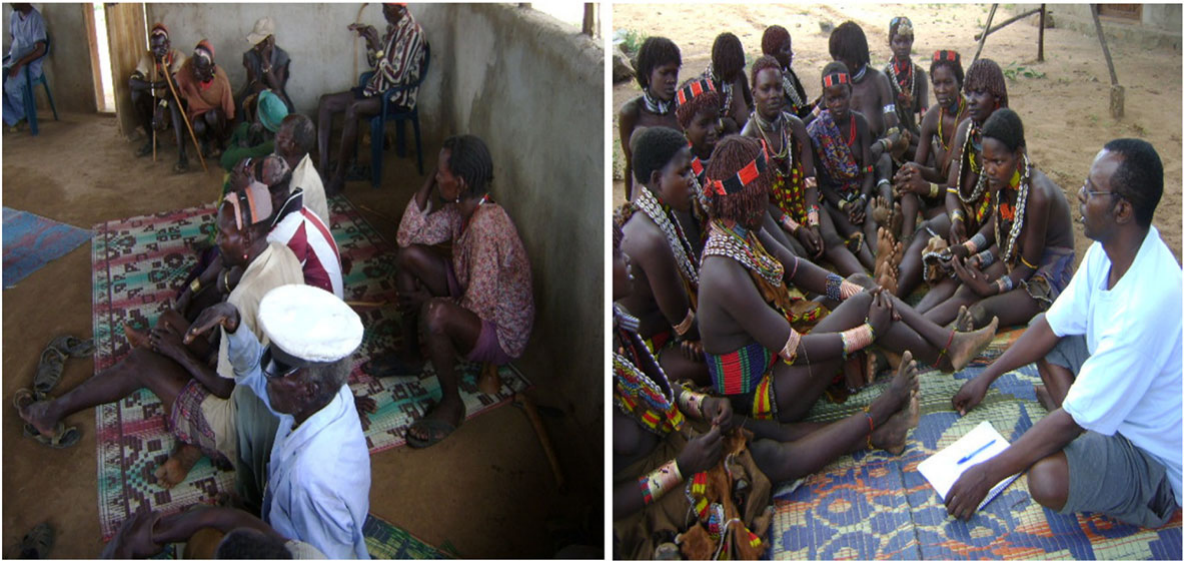
Focus group discussions

All participants underscored that the prevalence of most of these illnesses was high during the months of December, January, February and March where drought and shortage of water become the main challenges. The majority of the participants indicated that large number of community members go to traditional healers when they are sick. The participants underlined that traditional healers are capable of treating diseases with minimum cost and high reliability. In addition, FGD participants (Fig. 2) said that geographical accessibility and cultural acceptability have made traditional healers to be the most favored health care options.

In contrast, five participants from three different kebeles argued that modern medicine is their first choice if and when they seek medical care. They stated that health institutions deliver health care service following proper and evidence-based diagnosis better than traditional healers. Two participants from *Lala* kebele suggested that homemade remedies should be tried and their effectiveness proven before using them for medication.

According to the majority of FGD participants, plants are the major sources of TM among the Hamer ethnic group. Even though they were not keen to give details of these medicinal plants, they reported that they use a large number of plants to treat a variety of diseases. They underlined that the names and other details of these medicinal plants should only be disclosed to “special” persons. However, one of the participants explained that collecting medicinal plants on the basis of their colour was a very common pattern. For instance, plants such as *fulante* (*Dichrostachys cinerea* (L.) Wight & Arn.) and *guci* (*Lagenaria siceraria* (Monila) Standl.), which have pale yellow flowers are used for the treatment of jaundice (*ara*). It was observed that no special attention is given to plants with medicinal values and that they are treated just like any other plant. According to the participants, the main reason for this may be associated with the fact that pastoralists are usually on the move and are, therefore, unable to grow or cultivate plants on a regular basis. The majority of medicinal plants are collected from the wild. In addition to plants, animals and minerals are also used as a source of medicine for TM. Examples of such animal products include goat meat (*Qoli*), fats, milk and blood of goat and cow, and bone of goat.

The majority of the FGD participants claimed that knowledge of traditional medicines, particularly herbal medicine, is handed down from elders to younger generation through word of mouth. Among the Hamer ethnic group, traditional knowledge is transmitted only to the eldest son or a male member from close relatives. Otherwise, knowledge and skill of healing are held always in secret. Although the participants were aware of the menace of this type of transferring traditional knowledge and practice, they still believe that it should continue to preserve the dignity of their ancestors. In the case of knowledge transfer of *Merankal*, which is associated with divine power, the practitioners carry out rituals where their spirits tell them as to who should be their successor and ask their spirit to transfer their spiritual power to their successor. There were two FGD participants from *Lala* kebele with different views. According to them, healers are selected by nature and god (*Burjo*) to keep the well-being of their community.

Nearly all participants from *Ariya Kayusa, Achi Musa, Besheda* and *Shanko Kelema* kebeles expressed that young members of their community have much less interest in traditional medicine. Improved physical access to modern health institutions, the effect of modernization that comes through expansion of modern education and the Christian religion were mentioned by participants as the main reasons for such decline in interest by the younger generation.

### Key informants

A total of 8 respondents (seven males and one female) who belong to members of traditional healers of the community were interviewed. Five of the healers were nonliterate, and only three of the healers had received formal education.

### Illnesses treated, methods of diagnosis and sources of medicine

The categories of illnesses claimed to be treated by traditional medical practitioners varied from common infections to complicated conditions. The most frequently treated illnesses by traditional healers were *zen*, *ara, bishi burka*, *lognagena*, *gebez*, *chaki* and *guni* (Table 1). According to the traditional healers, visual observation and history taking were the two main methods of diagnosis. Spiritualism could also be employed whenever the patient condition is suspected to be due to spiritual afflictions, which could not be diagnosed by physical assessment and history taking.

**Table 1:** Illnesses and symptoms reported to be treated by traditional healers of the Hamer ethnic group, South Omo Zone (January–February 2010)

Category	Illness/symptoms
Infectious	*Silito* (tuberculosis), *gebez* (malaria), *zen* (diarrhoea), fever, tinea, *ara* (jaundice), *berdate* (intestinal worms), *astiajim* (toothache), *gulfadhana* (common cold), dysentery, *sal* (cough), wounds, tonsillitis
Non-infectious	*Lognagena* (blood pressure), *chaki* (evil eye), *guni* (snake bite), asthma, male sexual impotence, muscle pain, pain associated with menstrual cycle, *meta ajim* (migraine), nature spirits, curses, fractures

Those written in italics are local name of the disease

### Mode of service delivery

The present survey indicated that all of the healers provide their medical services on part-time basis. The majority of these practitioners had healing experience of less than 20 years. While delivering their services, nearly all of the traditional healers reported that they had a single assistant except for one practitioner who said that he had three assistants. Assistants are mostly healers’ own sons or male close relatives. None of the interviewed healers set costs for their service. They charge their customers after assessing their livelihood, and also on the basis of their relationship to the healer.

### Household survey

#### Perceived illnesses and action taken during the 2 weeks recall period

At the time of the survey, a total of 8523 individuals were living in the studied HHs, and during the 2 weeks recall period, 912 illness episodes were reported to have occurred; making prevalence rate of 10.7 %. Age distribution of individuals with reported illnesses shows that 458 (50.2 %) were ≤ 15 years old (Table 2). The survey indicated that 861 (94.4 %) of those who reported illness took action. Of these, 486 (53.3 %) went to healthcare facilities, 255 (27.9 %) went to traditional healers and 120 (13.2 %) used homemade remedies. Fifty-one persons with reported illness (5.6 %) did not take any action in response to their perceived illness symptoms (Table 2). The percentage of females who did not take action in response to the perceived illnesses during the 2 week recall period was slightly higher than males, 6.2 and 5.1 %, respectively.

**Table 2:** Actions taken against perceived illnesses during a 2 week recall period among the Hamer ethnic group, South Omo Zone (January-February 2010)

Demographic characteristics	Action taken N (%)				
	Went to health institutions	Went to traditional healers	Took homemade remedies	Took no action	Total
Sex					
Male	276 (58.2)	120 (25.3)	54 (11.4)	24 (5.1)	474 (52.0)
Female	210 (49.0)	135 (30.8)	66 (15.1)	27 (6.2)	438 (48.0)
Age					
≤ 5	168 (55.9)	34 (25.9)	35 (14.2)	10 (4.1)	247 (27.1)
5–15	146 (61.3)	23 (10.9)	4 (1.9)	38 (18.0)	211 (23.1)
15–65	171 (37.9)	196 (43.5)	81 (18.0)	3 (0.7)	451 (49.5)
≥65	1 (33.3)	2 (66.7)	-	-	3 (0.3)
Income status					
Low	279 (61.1)	125 (49.0)	74 (61.7)	37 (72.5)	533 (58.4)
Medium	123 (25.3)	87 (43.1)	39 (32.5)	9 (17.6)	258 (28.3)
High	66 (13.6)	43 (16.0)	7 (5.8)	5 (9.8)	121 (13.3)
Total	486 (53.3)	255 (28.0)	120 (13.2)	51 (5.6)	912 (100)

Low income: less than five cattle, no goats and hives; Middle income: five to ten cattle, goats and hives; High income: more than ten cattle, goats and hives

### Factors influencing actions taken and preferred health care options

The demographic and socio-economic status of the respondents with respect to choice of health care options, as well as actions taken against perceived illnesses are shown in Tables 2 and 3. It can be seen that, the percentage of those who took no action decreased from 72.5 to 9.8 % from low to high income groups, respectively (Table 2), indicating that economic status of HHs in the study group has significant effect on the actions taken against perceived illnesses (*χ* ^2^ = 11.988, df = 4, *P* < 0.05). The results also show that the majority of males had taken action. Thus, effect of sex on action taken in response to the symptoms of perceived illnesses during the 2 week recall period was found to be statistically significant (*χ* ^2^ = 9.677, df = 2, *P* < 0.05).

**Table 3:** Choices of healthcare options with respect to socio-demographic characteristics of household (HH) respondents among members of the Hamer ethnic group, South Omo Zone (January–February 2010)

Variable	Choice of health care N (%)			Total
	Health institution	Traditional healers	Homemade remedies	
Sex				
Male	380 (38.8)	434 (44.3)	165 (16.9)	979 (100)
Female	193 (31.1)	140 (22.5)	288 (46.4)	621 (100)
Age				
≤20	91 (35.5)	148 (57.8)	17 (6.7)	256 (100)
21–30	245 (45.0)	213 (39.2)	86 (15.8)	544 (100)
31–40	174 (35.1)	209 (42.1)	113 (22.8)	496 (100)
41–50	96 (40.0)	103 (42.9)	41 (17.1)	240 (100)
51–60	9 (19.1)	22 (46.8)	16 (34.1)	47 (100)
≥61	1 (5.9)	7 (41.2)	9 (52.9)	17 (100)
Educational status				
Nonliterate	480 (34.9)	505 (65.1)	391 (28.4)	1376 (100)
1–4 Grade	74 (38.5)	61 (31.8)	57 (29.7)	192 (100)
5–8 Grade	19 (59.4)	8 (25.0)	5 (15.6)	32 (100)
Family size				
1-3	190 (41)	198 (42.7)	76 (16.4)	464 (100)
4–6	207 (32.3)	243 (38.0)	190 (29.7)	640 (100)
7–9	133 (39.6)	76 (22.6)	127 (37.8)	336 (100)
≥10	43 (26.9)	57 (35.6)	60 (37.5)	160 (100)
Monthly income				
Low	295 (31.6)	402 (42.9)	238 (25.5)	935 (100)
Middle	162 (35.8)	101 (22.3)	190 (41.9)	453 (100)
High	116 (54.7)	71 (33.5)	25 (11.8)	212 (100)
Total	573 (35.8)	574 (35.9)	453 (28.3)	1600 (100)

Nonliterate: member of the community who can read but not write; Low income: less than five cattle, no goats and hives; Middle income: five to ten cattle, goats and hives; High income: more than ten cattle, goats and hives

The effect of age on action taken against perceived illnesses during the 2 weeks recall period was found to be significant (*χ* ^2^ = 170.485, df = 2, *P* < 0.0.05). Accordingly, the proportion of children for whom action was taken against perceived illnesses during the 2 weeks recall period was higher as compared to those with age above 15 years.

In terms of preference to choice of health care options, the percentage of those HH respondents who favored biomedical care in case of illness increased from 31.5 to 54.7 % with low and high income respondents, respectively. Likewise, those who chose TM as a first line option declined from 68.4 to 45.3 % in these groups. The effect of economic status on the choice of health care options of HH respondents was found to be statistically significant (*χ* ^2^ = 40.347, df = 2, *P* < 0.05). The influence of education on choice of treatment options of HH respondents was also statistically significant (*χ* ^2^ = 7.210, df = 1, *P* < 0.05). In this regard, literates (41.5 %) prefer biomedical care to TM as a choice of health care more than nonliterates (34.9 %) (Table 3).

### Plants reported to be in use

A total of 60 different medicinal plants were reported [34 by HH respondent, 14 by traditional healers and 12 by both]. Fifty-one (85 %) medicinal plants were fully identified, 3 (5 %) were identified at generic level and 6 (10 %) have not yet been identified.

Of the collected medicinal plants, the majority (85.2 %) are used for treating human diseases, 6.6 % for veterinary diseases and 8.2 % for both human and veterinary diseases (Table 4).

**Table 4:** Commonly treated illnesses with herbal remedies in household (HH) and by traditional healers among the Hamer ethnic group (January–February 2010)

Category	Indications	Frequency
Skin/dermatological problems	Skin allergies (143), wounds (154), snake/scorpion bites (419), dandruffs (85), eczema (73), burns (112), tumors of skin/abscess (98), fungal skin infection (44), tinea capitis (162), skin rash (itching) (41)	1087
Abdominal and GIT problems	Diarrhoea (178), abdominal colic (267)*,* abdominal discomfort (141)	586
Liver diseases	Jaundice (426)	426
Respiratory tract problems	Common cold (218), dry cough (123)	341
Parasitic infections	Malaria (518), intestinal helminths (432)	950
ENT (Eye, nose and throat)	Eye diseases (274), toothache (463), tonsillitis (116)	853
Cardiovascular problems	Hypertension (27)	27
Others	Evil eye (303), muscle and joint pain (121), headache (118), loss of appetite (1), dysmenorrhoea (1), irregular menses (1)	545

Numbers in brackets indicate the number of respondents claimed to use the medicinal plants for that specific illness

**Fig. 3:**
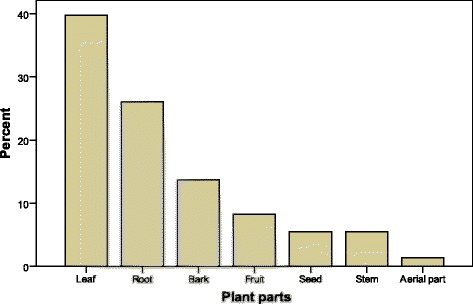
Plant parts used in the preparation of herbal remedies among the Hamer ethnic group, January - February, 2010

The identified plants belonged to 27 families. Among the families, Fabaceae is the most commonly reported family which comprised seven species followed by Solanaceae (six), Combertaceae and Capparidaceae (each three) (Tables 5, 6 and 7). The most common morphological parts used for the preparation of herbal remedies are leaves (38.0 %), roots (26.6 %) and barks (13.9 %) (Fig. 3).

**Table 5:** Medicinal plants reported by household (HH) respondents of the Hamer ethnic group, South Omo Zone (January–February 2010)

Vernacular name	Scientific name (Collection number)	Family	Part(s) used	Medicinal indication(s)	Method of preparation and use
Chaki Dhesha	*Barleria eranthemoides* R. Br. ex C. B. Clarke (H022)	Acanthaceae	Leaf	Evil eye	Pounded, boiled with water, filtered and drunk
Kufuri	*Rhus natalensis* Krauss^N^ (H040)	Anacardiaceae	Fruit	Various disease of stomach	Macerated in water, filtered, mixed with honey, and drunk
			Stem	Malodor of mouth	Gently chewed for about an hour
Mordhe	*Launaea intybacea* (Jacq.) Beauv.^N^ (H046)	Asteraceae	Root	Abscess	Ground, macerated in water, and the filtrate applied on the affected area
Dhumuko	*Balanites aegyptiaca* (L.) Del.^N^ (H025)	Balanitaceae	Bark	Hypertension	Inside part of the bark peeled off, boiled with water, filtered and drunk
Alela	*Boswellia neglecta* S. Moore^N^ (H044)	Burseraceae	Exudate	Evil eye	Dried, burned and the smoke inhaled
Beles	*Opuntia ficus-indica* (L.) Miller (H059)	Cactaceae	Leaf	Hair loss	Sliced and rubbed against the affected part of the scalp
Zegurma	*Combretum aculeatum* Vent^N^ (H034)	Combertaceae	Leaf	Abdominal colic	Fresh leaves chewed, and juice swallowed
Ara	*Treminalia brownii* Fresen.^N^ (H038)	Combretaceae	Bark	Jaundice for both human and animals	Inner bark peeled, chopped, macerated in water, filtered and drunk
Wefenkur	*Commelina benghalensis* L. (H054)	Commelinaceae	Exudate	Skin problem	Applied on the affected area
Gusi	*Lagenaria siceraria* (Monila) Standl. (H060)	Cucurbitaceae	Fruit	Jaundice	Fruit dissected and patient’s face covered with the inside part of the dissected fruit
Busente	*Cyperus alternifolius* L^N^ (H055)	Cyperaceae	Root	Abdominal colic	Chopped, chewed, and juice swallowed
Alko/Algi	*Sansevieria ehrenbergii* Schweinf. ex Baker ^N^ (H018)	Dracaenaceae	Leaf	Wound healing	Fresh leaves pounded, and juice applied on wound
			Root	Muscle pain	Fresh root chopped, boiled in water, filtered and drunk
Atmin Dhesha	*Sansevieria forskaoliana* (Schult.f.) Hepper & Wood (H020)	Dracaenaceae	Leaf	Blister after burning	Fresh leaves smashed, juice applied on the site of burning
Kera	*Euphorbia* sp. (H027)	Euphorbiaceae	Bark	Hypertension	Fresh bark chopped, macerated in water, filtered, mixed with honey and drunk
Sewute	*Acacia tortilis* (Forssk.) Hayne (H043)	Fabaceae	Leaf	Goat intestinal parasite	Fresh leaves fed to goats
Dhita	*Albizia anthelmintica* (A.Rich.) Brogn. (H009)	Fabaceae	Bark	Intestinal parasite	Inside part of the fresh bark cut, boiled with water, filtered, mixed with sorghum powder and eaten
Chaqidhesha	*Indigofera* sp. (H036)	Fabaceae	Root	Evil eye	Chewed and juice swallowed
Moshke	*Ormocarpum trichocarpum* (Taub.) Engl. (H031)	Fabaceae	Leaf	Abscess	Chopped, macerated in water and applied on swollen skin
Armacha	*Senna italica* Mill.^N^ (H017)	Fabaceae	Leaf	Allergy on skin	Fresh leaves crushed, stood in cold water and filtrate drunk
Bishidhesha	*Ocimum lamiifolium* Hochst. ex Benth.^N^ (H050)	Lamiaceae	Leaf	Skin diseases	Crashed and rubbed on affected area
Gudemburkanane	*Plectaranthus* sp. (H037)	Lamiaceae	Leaf/Root	Abdominal colic	Leaves or roots chopped, boiled with water and decoction drunk
Chursha	*Sida rhombifolia* L.^N^ (H048)	Malvaceae	Aerial part	Bone strength	Fresh aerial part ground, macerated in water, and filtrate drunk
Dhare/Fire	*Cissampelos pariera* L. (H023)	Menispermaceae	Leaf	Wound healing	Fresh leaves squeezed on wound
Kelewa	*Rhamnus prinoides* L’Herit ^N^ (H058)	Rhamnaceae	Fruit	Skin diseases	Macerated in water and the swollen fruit rubbed against the affected skin
Medhel	*Canthium pseudosetiflorum* Bridson (H032)	Rubiaceae	Leaf	Malaria	Ground, macerated with water, filtered and drunk
Kena	*Vepris glomerata* (F. Hoffm.) Engl.^N^ (H029)	Rutaceae	Bark/Leaf	Malaria, abdominal colic	Fresh leaves or mixed with bark cut into pieces, stood in water, filtered and drunk
Gedeqa	*Zanthoxylum chalybeum* Engl. ^N^ (H026)	Rutaceae	Fruit	Abdominal discomfort	Dried fruits roasted, chewed and swallowed
Kerja	*Salvadora persica* L.^N^ (H028)	Salvadoraceae	Root/Stem	Gum bleeding	Root or stem chewed, and juice kept in the mouth
Meta dhesha	*Datura stramonium* L. (H042)	Solanaceae	Leaf	Tinea	Fresh leaves chopped, squashed, and juice applied on scalp
Gerante	*Solanum dasyphyllum* Schumach.^N^ (H049)	Solanaceae	Root	Abdominal colic	Chopped, chewed, and swallowed
Butambero	*Withania somnifera* (L) Dunal ^N^ (H021)	Solanaceae	Root	Common cold, tonsillitis	Fresh roots chewed and juice swallowed
Gergesho	*Grewia villosa* Willd^N^ (H039)	Tialiaceae	Fruit	Intestinal parasite	Chewed and swallowed

^N^: Native to Ethiopia

**Table 6:** Medicinal plants reported by traditional healers among the Hamer ethnic group (January–February 2010)

Vernacular name	Scientific name	Family	Part(s) used	Medicinal use(s)	Method(s) of preparation
Busente	*Hypoestes forskaolii* (Vahl) R.Br. (H052)	Acanthaceae	Root	Evil eye	Fresh roots ground, macerated in water, filtered and drunk
Zen dhesha	*Amaranthus hybridus* L. (H053)	Amaranthaceae	Seed	Diarrhoea	Powdered, cooked in water and drunk with honey
Ekumangenta	*Amaranthus spinosus* L. (H045)	Amaranthaceae	Root	Toothache	Crushed, and pressed on tooth
Dhela	*Cadaba farinosa* Forssk.^N^ (H024)	Capparidaceae	Root	Hypertension	Chopped, boiled with meat soup and drunk
				Malaria	Chopped, boiled with meat soup and drunk
-	*Maerua triphylla* A. Rich (H011)	Capparidaceae	Root	Irregular menstruation, loss of appetite	Crushed, stood in water, filtered, and drunk
Bote	*Cucurbita pepo* L (H035)	Cucurbitaceae	Seed	Intestinal parasite	Dried, roasted, chewed and swallowed
Dhenqesho	*Zehneria pallidinervia* (Harms) C. Jeffery (H014)	Cucurbitaceae	Leaf	Cattle eye disease	Fresh leaves crushed, and juice instilled into the affected eye
Gebezdhesha	*Cyperus distans* L.f. (H004)	Cyperaceae	Bark/Root	Malaria	Roots or mixed with inner bark, chopped, macerated in water, mixed with milk and drunk
Fulante	*Dichrostachys cinerea* (L.) Wight &Arn.^N^ (H010)	Fabaceae	Bark	Jaundice	Inner part peeled off, chopped, boiled with water smoke is inhaled
Lalombe aka	*Leucaena leucocephala* (Lam.) De Wit (H013)	Fabaceae	Stem	Hypertension, intestinal parasite, irregular menstruation, loss of appetite	Chopped, macerated, filtered, mixed with honey and milk, and drunk
Choq	*Chasmanthera dependens* Hoscht. (H041)	Menispermaceae	Leaf	Toothache	Fresh leaves chopped, smashed, and pressed on tooth
Butambero	(H006)	-	Leaf/Root	Shello/Bishifechi	Chopped, macerated in water, filtered and drunk
Buri	(H008)	-	Root	Abdominal colic	Crushed, infusion prepared, filtered, mixed with milk and drunk
				Evil eye	Burnt, and smoke inhaled
Adema	(H019)	-	Bark	Ara	Chopped, boiled in water and vapor inhaled
Onoko	(H057)	-	Leaf	Koliberdate	Fed to goats

^N^: Native to Ethiopia

**Table 7:** Medicinal plants reported by traditional healers and HH respondents of the Hamer ethnic group, South Omo Zone (January–February 2010)

Vernacular name	Scientific name (Collection number)	Family	Part(s) used	Medicinal use(s)	Method (s) of preparation
Welqante	*Aloe otallensis* Baker^N^ (H002)	Aloaceae	Exudate	Malaria	Mixed with honey and milk, and drunk
				Wound healing	Applied on wound
Salbana	*Ozoroa insignis* Del. (H005)	Anacardiaceae	Bark	Malaria	Inner part peeled off, chopped, macerated in water, filtered and drunk
Gebez Dhesha	*Adenium obesum* (Forssk.) Roem. & Schult.^N^ (H003)	Apocynaceae	Root	Abdominal colic	Chopped, chewed and juice swallowed
				Evil eye	Chopped, boiled in water, cooled and drunk
Akemba	*Carissa spinarum* L (H016)	Apocynaceae	Root	Malaria	Crushed, infusion prepared in water, filtered, and drunk
Feto	*Lepidium sativum* L. (H056)	Brassicaceae	Seed	Allergic reaction on skin, ulcer in mouth and throat	Ground, mixed with butter, applied on the skin, mouth and throat
Menzo	*Cadaba mirabilis* Gilg. (H030)	Capparidaceae	Leaf	Cattle disease	Crushed and juice instilled into the affected eye
Aradhesha	*Maytenus senegalensis* (Lam.) Excell (H047)	Celasteraceae	Leaf	Jaundice	Chopped, boiled in water and vapor inhaled
Tuzi	*Euphorbia tirucalli* L.^N^ (H033)	Euphorbiaceae	Leaf/Stem	Wound healing	Fresh juice applied on wound
Kelanqi	*Moringa stenopetala* (Bak. f) Cuf.^N^ (H051)	Moringaceae	Leaf	Hypertension/Abdominal colic	Fresh leaves boiled, allowed to cool, and the filtrate drunk
			Leaf/Root	Malaria	Fresh leaves or roots or both boiled, allowed to cool, filtered, mixed with honey and drunk
Guni Dhesha	*Datura metel* L. (H007)	Solanaceae	Leaf	Snake bite	Fresh leaves squashed and juice applied on the affected area
Gerante	*Solanum incanum* L.^N^ (H001)	Solanaceae	Fruit	Wound healing	Ripen fruit squeezed on wounds
			Root	Toothache	Chewed and juice swallowed
Gurdo/Ardo	(H015)	-	Leaf	Snake bite	Fresh leaves chopped, squashed, and juice applied on the affected area

^N^: Native to Ethiopia

Among the collected plants, 68.9 % were from forests or wild sources and 13.1 % were cultivated or garden plants, and 18 % were obtained from both forests and gardens. The most widely used preparation methods include maceration, decoction and infusion. The majority of the preparations are simple recipes (using only one plant as ingredient), while one of the preparations contained mixture of plants.

**Fig. 4:**
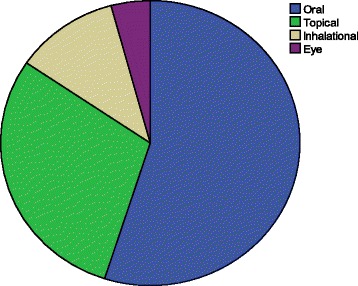
Routes of administration of herbal remedies among the Hamer ethnic group, January - February, 2010

The vast majority of the recipes were taken orally (54.9 %), followed by topical (29.6 %), inhalation (11.3 %) and instillation into the eye (4.2 %) (Fig. 4). According to the current survey, most of the preparations were single dose preparations but the dosages were poorly established. Respondents of both HHs’ survey and traditional healers reported that vomiting, headache, diarrhoea, abdominal colic and irritation are the most common side effects of herbal preparations mentioned by the respondents and healers.

## Discussion

The findings of this study indicated that the majority of the healers are males. Although the number of key informants in this survey was small, similar trends were found in previous surveys [10–14]. This higher number of male traditional healers than females in traditional healing practice might be due to the tradition of the healing practice that does not encourage women to be involved in. The majority of the plants used to treat diseases are collected from the wild. Thus, considering the time and effort required to collect these materials together with other socio-cultural factors such as transfer of knowledge from elders to male members of the family in secret, discourage women to be actively involved in the practice of traditional medicine [10, 13].

According to the traditional healers, visual observation and history taking were the two main methods of diagnosis. Spiritualism could also be employed whenever the patient condition is non-natural causes which could not be diagnosed by physical assessment and history taking. The sources for healing constitute different components of traditional medical practices. The results of the present survey indicated that the majority of healers used plant and animal products to treat illnesses, while two of them used animal products combined with spiritual power, and only one used spiritual power to treat illnesses. In addition to these, some traditional healers perform minor surgeries.

In agreement with the present study, other similar studies indicated that many traditional healers practice on a part-time basis but that they have a long experience in the profession [10, 11, 15]. It has been observed that long experience is needed for traditional medicine practitioners (TMPs) to be effective [15].

Similar to the results obtained from elsewhere [14], traditional healers in the Hamer ethnic group reported that they use their sons or male close relatives as an assistant. In most cases the responsibilities of the assistants were limited to preparing equipment and materials required for treatment of patients. In addition, they help weak clients who are unable to take care of themselves. In some cases when the healer is too old, they carry out his duties as per his instructions and under his supervision.

None of the interviewed healers had fixed payment rate for their services. The rate is determined on the basis of customer’s economic condition and relation to healers. A previous survey carried out in other parts of Ethiopia also documented a similar finding [10].

Concurrent with the report generated from other studies [16, 17], the present study indicates that a high proportion (64.2 %) of HH respondents reported to have sought help from TMPs. The Hamer ethnic group largely tended to seek help from TMPs for the following reasons; firstly, they are nomads/pastoralists and therefore move from one place to another following the track of their cattle. Secondly, the majority of community members live in poverty and poor infrastructure. Thirdly, the cost of traditional medicine is very low compared to modern drugs and this is compounded with the cultural beliefs of the community that only traditional medicine is effective in combating certain types of illnesses such as evil eye (*chaki)*, snake bite (*guni*) and hypertension (*lognagena*)*.* In addition, factors such as lack of information and community members’ desire for health services that are readily available, affordable and socially and culturally acceptable, play a decisive role for their choice [18]. Hence, TM remains the mainstay in narrowing the gap of their health care needs [17, 19, 20]. These results obtained from HH respondents concur with the findings of FGDs.

Patterns of health service utilization and health care seeking were found to be influenced by socio-economic status, level of education, cultural beliefs and perceptions of the causes of diseases and scope for treating different conditions [18, 19, 21]. In this study females (69 %) sought more help from TMPs than males (61.1 %) for their health care. These results are in agreement with a previous study carried out in Ethiopia [2] but different from surveys conducted in other countries [19, 22, 23]. This might be due to the enormous burden on females in the Hamer ethnic group to look after the family; long distances form health institutions and poor infrastructure, low income status and the cultural beliefs of the community [18, 24, 25].

The influence of education on choice of treatment options of HH respondents was statistically significant (*χ* ^2^ = 7.210, df = 1, *P* < 0.05). For that reason, literates (41.5 %) prefer health care facilities to TM as a choice of health care more than nonliterates (34.9 %). Thus, the present survey clearly showed that income status and educational status of HH respondents could influence the choice and quality of health care needs and actions taken against perceived illnesses. Respondents with higher economic status and literates sought modern health care services more than those with lower economic status and nonliterates (*P* < 0.05). Similar trends have been demonstrated in previous studies [17, 19, 26]. The findings appear to indicate that people with lower socio-economic status might have problems of access to modern health care facilities as they may not afford the cost [21], and/or lack of education could also impinge on the awareness of the community members about the ailments and seeking help for health care [18, 19].

In terms of preference to choice of health care options, the percentage of those HH respondents who favored health institutions in seeking medical care when a family member gets sick rose from 31.5 to 54.7 % from low income respondents to high income respondents. Likewise, those who chose TM as a first line declined from 68.4 to 45.3 % in these groups. The effect of economic status on the choice of health care options of HH respondents was found to be statistically significant (*χ* ^2^ = 40.347, df = 2, *P* < 0.05).

Of the collected and identified medicinal plants, Fabaceae is the most commonly reported family, which is in agreement with other surveys carried out in different parts of the country [27]. This is not surprising as Fabaceae is the second largest family in the country behind Asteraceae, in addition to being among the most common families found in dry forests [27]. In this study, a large number of medicinal plants are collected from the wild, a finding similar with surveys conducted in other parts of Ethiopia [26, 28]*,* Kenya [29], Ghana [15], Brazil [30], Serbia [31], and Malaysia [32]. However, at least in one survey conducted in Northern Ethiopia [33], the majority of medicinal plants are collected from gardens. In general, collection of medicinal plants from forests indicates that there is little practice of preserving medicinal plants in cultivated areas or home gardens. In the context of the current survey, the reason could be associated with the life style of the community, who are by and large pastoralists. This, together with poor protection of wild medicinal plants due to the ongoing mass destruction of wild vegetation for different purposes by the community and overgrazing are endangering medicinal plants and discourage the practice of traditional health care in the study area [15, 20, 28].

The main reasons for the most common use of leaves and roots could be due to the fact that they act as reservoirs for exudates/secretions which are believed to contain toxins, some of which may have medicinal value, and also due to the relative ease of finding these plant parts [34, 35]. The popularity of roots as a source of herbal drugs has serious consequences from both ecological point of view and the survival of the medicinal plant species [36]. Therefore, due attention must be given to this problem before the situation gets worse.

The majority of the preparations are simple recipes (using only one plant as ingredient), while one of the preparations contained a mixture of plants. The use of simple recipes has been reported in other parts of the world [30, 37]. The combination of more than one plant in herbal preparations could increase the potency mainly due to synergistic or additive effect. Whilst the majority of the remedies were prepared form freshly collected plant parts, dried parts are also used to prepare very few plant drugs, a finding that was consistent with other works conducted in Ethiopia [20, 38], India [37] and Brazil [30]. The possible justification for the use of fresh plant material could be due to the simplicity of the method which does not require sophisticated equipments.

## Conclusion

The present study revealed that health seeking behavior of the Hamer ethnic group is affected by different socio-economic and cultural factors. There is also a strong indication for traditional medical practices and use of plant materials to treat various ailments and health problems among the study population. Selection of medicinal plants by the Hamer ethnic group appears to have sound basis as most community members claim to have benefited from the use of herbal drugs. The study also showed that the majority of medicinal plants are collected from the wild; with leaves and roots being the most widely used plant parts. Collection of leaves from wild may not pose a serious danger to the survival of a plant. However, collection of roots may contribute to the destruction of the plant species causing high risk of loss of biodiversity. The use of medicinal plants in particular, and traditional medicine in general, among the study population is facing danger of survival as the means of transferring knowledge form one generation to another is mainly by word of mouth, and the younger generation appears to have no interest in acquiring such knowledge. Therefore, it is important that the government creates awareness among community members about the significance of preserving traditional knowledge and also conserving medicinal plants before they disappear. Furthermore, additional surveys on other minority ethnic groups of the woreda should be conducted in order to collect, identify and document medicinal plants and other traditional medical practices.

## Additional file

Additional file 1: Appendix I Glossary of local names of illnesses and some traditional medical practices and their equivalent meanings in English. Appendix II Questionnaire to be used to collect ethnopharmacological information at house hold level among Hamer ethnic group, Hamer Woreda, South Omo Zone, SNNPR. Appendix III Questionnaire to be used to collect ethnopharmacological information for key informants among Hamer ethnic group, Hamer Woreda, South Omo Zone, SNNPR. Appendix IV Questions for Focused Group Discussions. (DOCX 24 kb)
