# Supplementary material for: Health seeking behavior and use of medicinal plants among the Hamer ethnic group, South Omo zone, southwestern Ethiopia
Source: J Ethnobiol Ethnomed. 2016 Oct 6;12:44. doi: 10.1186/s13002-016-0107-x (PMC5052888; doi:10.1186/s13002-016-0107-x)
Supplement: Additional file 1: — Appendix I Glossary of local names of illnesses and some traditional medical practices and their equivalent meanings in English. Appendix II Questionnaire to be used to collect ethnopharmacological information at house hold level among Hamer ethnic group, Hamer Woreda, South Omo Zone, SNNPR. Appendix III Questionnaire to be used to collect ethnopharmacological information for key informants among Hamer ethnic group, Hamer Woreda, South Omo Zone, SNNPR. Appendix IV Questions for Focused Group Discussions. (DOCX 24 kb) [file 13002_2016_107_MOESM1_ESM.docx]

**Additional file 1**

**Appendix I** Glossary of local names of illnesses and some traditional medical practices and their equivalent meanings in English.

*Afi Burka*: Illness characterized by itching, redness and pain to the eye

*Ara*: Illness which appear as yellow colour of scelera of the eye, nail, skin (symptoms similar to jaundice)

*Berdate*: Fungal infection of the surface skin of the face (similar to tinea faciei)

*Bishi Ajim*: Illness of the skin usually infectious like fungal infections

*Chaki*: Evil eye

*Dedmedmat*: Bleeding of jinjiva

*Dunguri Qansa*: A superstitious belief of predicting the future by measuring heel drop

*Emburikadana*: Illness which is characterized by abdominal spasm/colic

*Ethadhana*: Illness characterized by swelling of the skin

*Fersi*: Traditional alcoholic drink made of sorghum and corn

*Gebeze*: Illness characterized by fever, chilling, loss of appetite tiredness shivering (symptoms similar to malaria)

*Gulfadhana*: Common cold

*Guni*: Severe pain caused by the bite of snake/scorpion

*Kolin Ashedha*: Looking at goat intestine for predicting the future

*Korokor*: Fungal infection of the scalp

*Logagna*: Illness characterized by headache, lightheadedness, pounding in the neck and fatigue (symptoms similar to hypertension)

*Merankal*: Traditional practice which involve witch-hunt

*Shello Fecha*: Itchy and redness of skin (symptoms similar to inflammatory skin disorders)

*Shoforo*: Traditional coffee made from coffee peel

*Zen*: Dioarrhea.

**Appendix II** Questionnaire to be used to collect ethnopharmacological information at house hold level among Hamer ethnic group, Hamer Woreda, South Omo Zone, SNNPR.

Date of Interview________________ Time____________________

Name of Interviewer ______________________________

1. Address of Respondent

Kebele_________________ Village____________

Instruction: Circle the Letter that Contain the Appropriate Choice

2. Demographic Information

2.1 Sex: 1. Male 2. Female

2.2 Age_________

3. Religion

1. Orthodox 2. Muslim 3. Protestant 4. Others, if any___________________

4. Status of the family

1. Husband 2. Wife 3. Head of Household (Woman) 4. Others

5. Household size_______________

6. Occupation__________________

7. Education Status

1. Illiterate 2. Religious education 3. Basic education 4. Grade 1-4

5. Grade 5-8 6. Grade 9-10 7. Grade 11-12 8. Above 12, __________

8. Economic Status

8.1 Type of House: A. Permanent B. permanent

8.2 Average monthly income, if known____________

8.3 Number of Domestic animals:

Cattle__________ Goat/Sheep_____________ Horse/Donkey

8.4 Source of information

1. Radio 2. Television 3. Other, Specify______________

9. Was there any family member who gets ill in the past two weeks?

1. Yes_____________ 2. _______________

(If your answer is yes to question is 9, fill the table below)

| Sino | Sex | Age | Perceived illness (Symptoms) | What do you think the cause of illness | Taken action to alleviate the problem | | | |
| --- | --- | --- | --- | --- | --- | --- | --- | --- |
|  |  |  |  |  | Went to Health institution | Went to healer | Used homemade remedy | No action |
| 1 |  |  |  |  |  |  |  |  |
| 2 |  |  |  |  |  |  |  |  |
| 3 |  |  |  |  |  |  |  |  |
| 4 |  |  |  |  |  |  |  |  |

10. What do you do when any member of the family get sick?

1. Go to health institutions

2. Go to traditional healers

3. Use homemade remedies

4. Others, Specify_____________

11. What is your preference to choose the option in question 10?

1. It is cheap

2. It is more effective

3. No access to other alternative

4. Other, Specify______________

12. Do you know any plants used as medicines in your district? A. Yes B. No

(If your response to Q. 12 is yes, fill in the table below)

|  |  | Medicinal plants | | | |
| --- | --- | --- | --- | --- | --- |
|  |  | 1 | 2 | 3 | 4 |
| 12.1 | Vernacular name |  |  |  |  |
| 12.2 | Indications |  |  |  |  |
|  | 1 |  |  |  |  |
|  | 2 |  |  |  |  |
|  | 3 |  |  |  |  |
| 12.3 | Part of plant used |  |  |  |  |
|  | 1. Leaf |  |  |  |  |
|  | 2. Flower |  |  |  |  |
|  | 3. Bark |  |  |  |  |
|  | 4. Seed |  |  |  |  |
|  | 5. Root |  |  |  |  |
| 12.4 | Mode of Use |  |  |  |  |
|  | 1. Fresh |  |  |  |  |
|  | 2. Dry |  |  |  |  |
|  | 3. Both |  |  |  |  |
| 12.5 | Time of Collection |  |  |  |  |
|  | 1. Dawn |  |  |  |  |
|  | 2. Mid day |  |  |  |  |
|  | 3. Down |  |  |  |  |
|  | 4. Any time |  |  |  |  |
|  | Why time of collection is necessary? |  |  |  |  |
| 12.6 | Area of Growth |  |  |  |  |
|  | 1. Domestic(Cultivated) |  |  |  |  |
|  | 2. Wild |  |  |  |  |
|  | 3. Both |  |  |  |  |
| 12.7 | Precaution taken during Collection |  |  |  |  |
| 12.8 | Preparation of the medication |  |  |  |  |
|  | 1. Infusion |  |  |  |  |
|  | 2. Decoction |  |  |  |  |
|  | 3. Other, Specify |  |  |  |  |
| 12.9 | Dosage regimen |  |  |  |  |
|  | 1. Dose/ quantity |  |  |  |  |
|  | 2. Measurement used |  |  |  |  |
|  | 3. Frequency |  |  |  |  |
|  | 4. Duration of treatment |  |  |  |  |
| 12.10 | Route of Administration |  |  |  |  |
|  | 1. Oral |  |  |  |  |
|  | 2. Topical |  |  |  |  |
|  | 3. Inhalation |  |  |  |  |
|  | 4. Others, Specify_______ |  |  |  |  |
| 12.11 | Dietary items restricted through the course of therapy |  |  |  |  |
| 12.12 | Precaution during treatment |  |  |  |  |
| 12.13 | Adverse effects |  |  |  |  |
|  | 1. |  |  |  |  |
|  | 2. |  |  |  |  |
|  | 3. |  |  |  |  |
| 12.14 | Antidotes |  |  |  |  |
| 12.15 | Contraindications |  |  |  |  |
|  | 1. Children |  |  |  |  |
|  | 2. Geriatrics |  |  |  |  |
|  | 3. Pregnant |  |  |  |  |
|  | 4. Lactating mother |  |  |  |  |
|  | 5. Disease condition, Specify___________ |  |  |  |  |
| 12.16 | Shelf Life |  |  |  |  |
| 12.17 | Storage |  |  |  |  |
|  | 1. Container used |  |  |  |  |
|  | 2. Place of Storage |  |  |  |  |

**Appendix III** Questionnaire to be used to collect ethnopharmacological information for key informants among Hamer ethnic group, Hamer Woreda, South Omo Zone, SNNPR.

Date of Interview________________ Time____________________

Name of Interviewer ______________________________

1. Address of Respondent

Kebele_________________ Village____________

Instruction: Circle the Number that Contain the Appropriate Choice

2. Demographic Information

2.1 Sex: 1. Male 2. Female

2.2 Age_________

3. Religion

1. Orthodox 2. Muslim 3. Protestant

4. Others, if any______________________

4. Education Status

1. Illiterate 2. Religious education 3. Basic education 4. Grade 1-4

5. Grade 5-8 6. Grade 9-10 7. Grade 11-12 8. Above 12, __________

5. Economic Status

5.1 Type of House: A. Permanent B. permanent

5.2 Average monthly income, if known____________

5.3 Number of Domestic animals:

Cattle__________ Goat/Sheep_____________ Horse/Donkey___________

6. Source of information

1. Radio 2. Television 3. Other, Specify______________

7. Years of Experience as healer_______________________

8. Source of Knowledge and skills as healer

1. Family members

2. Religious institutions

3. Friends

4. Gift of Nature

5. Others, Specify________________

8. Manner of Practice

1. Full time 2. Part time

9. Do you have assistant(s) while practicing Healing? 1. Yes 2. No

10. If your answer to Q10, do you let it to observe all your healing practices? 1. Yes 2. No

11. Average Number of patients treated per day_____________

12. How much (on average) do you get monthly from your TM practice?

13. Do you have set treatment costs? 1. Yes 2. No

14. If yes for Q 13, how much is the average cost per medication? ______________

15. If not for Q13, How do charge your clients? ____________________________________

16. Illness treated

| Sino | Name of Illness | Symptom | Causes | Mode of transmission |
| --- | --- | --- | --- | --- |
|  |  |  |  |  |
|  |  |  |  |  |
|  |  |  |  |  |
|  |  |  |  |  |
|  |  |  |  |  |
|  |  |  |  |  |
|  |  |  |  |  |

17. What are your sources of medicines?

1. Plants

2. Animals

3. Minerals/Soil

4. Others, Specify______________

18. Would you mind telling plants which used as Medicine?

|  | | Medicinal plants | | | |
| --- | --- | --- | --- | --- | --- |
|  |  | 1 | 2 | 3 | 4 |
| 18.1 | Vernacular name |  |  |  |  |
| 18.2 | Indications |  |  |  |  |
|  | 1 |  |  |  |  |
|  | 2 |  |  |  |  |
|  | 3 |  |  |  |  |
| 18.3 | Part of plant used |  |  |  |  |
|  | 1. Leaf |  |  |  |  |
|  | 2. Flower |  |  |  |  |
|  | 3. Bark |  |  |  |  |
|  | 4. Seed |  |  |  |  |
|  | 5. Root |  |  |  |  |
| 18.4 | Mode of Use |  |  |  |  |
|  | 1. Fresh |  |  |  |  |
|  | 2. Dry |  |  |  |  |
|  | 3. Both |  |  |  |  |
| 18.5 | Time of Collection |  |  |  |  |
|  | 1. Dawn |  |  |  |  |
|  | 2. Mid day |  |  |  |  |
|  | 3. Down |  |  |  |  |
|  | 4. Any time |  |  |  |  |
|  | Why time of collection is necessary? |  |  |  |  |
| 18.6 | Area of Growth |  |  |  |  |
|  | 1. Domestic(Cultivated) |  |  |  |  |
|  | 2. Wild |  |  |  |  |
|  | 3. Both |  |  |  |  |
| 18.7 | Precaution taken during Collection |  |  |  |  |
| 18.8 | Preparation of the medication |  |  |  |  |
|  | 1. Infusion |  |  |  |  |
|  | 2. Decoction |  |  |  |  |
|  | 3. Other, Specify |  |  |  |  |
| 18.9 | Dosage regimen |  |  |  |  |
|  | 1. Dose/ quantity |  |  |  |  |
|  | 2. Measurement used |  |  |  |  |
|  | 3. Frequency |  |  |  |  |
|  | 4. Duration of treatment |  |  |  |  |
| 18.10 | Route of Administration |  |  |  |  |
|  | 1. Oral |  |  |  |  |
|  | 2. Topical |  |  |  |  |
|  | 3. Inhalation |  |  |  |  |
|  | 4. Others, Specify_______ |  |  |  |  |
| 18.11 | Dietary items restricted through the course of therapy |  |  |  |  |
| 18.12 | Precaution during treatment |  |  |  |  |
| 18.13 | Adverse effects |  |  |  |  |
|  | 1. |  |  |  |  |
|  | 2. |  |  |  |  |
|  | 3. |  |  |  |  |
| 18.14 | Antidotes |  |  |  |  |
| 18.15 | Contraindications |  |  |  |  |
|  | 1. Children |  |  |  |  |
|  | 2. Geriatrics |  |  |  |  |
|  | 3. Pregnant |  |  |  |  |
|  | 4. Lactating mother |  |  |  |  |
|  | 5. Disease condition, Specify___________ |  |  |  |  |
| 18.16 | Shelf Life |  |  |  |  |
| 18.17 | Storage |  |  |  |  |
|  | 1. Container used |  |  |  |  |
|  | 2. Place of Storage |  |  |  |  |

**Appendix IV** Questions for Focused Group Discussions

I would like to thank in advance all of you for your participation in group discussion.

1. What are the commonest illnesses in your society?

2. What do you usually do during illnesses?

3. What are the reasons for your Choice?

4. What are the major Sources of TM in your Society?

5. Is there any special attention given to plants used as medicine or treated in the same way as non-medicinal plants?

6. What are the trend of TM use in terms of availability of plants and acceptance by younger generation?

7. How is Knowledge of healing transferred from generation to generation?

8. What do you recommend to satisfy the health care needs of your society?

9. What are the Benefits and Limitation of Traditional medical practices to your community?
